# Impact of Active and Passive Maxillary Plates on Cleft Width Morphology in Unilateral Cleft Lip and Palate: A Prospective Intervention Study

**DOI:** 10.3390/children12060714

**Published:** 2025-05-30

**Authors:** Sarah Bühling, Helena Mariella Selge, Sara Eslami, Lukas Benedikt Seifert, Babak Sayahpour, Nicolas Plein, Robert Sader, Stefan Kopp

**Affiliations:** 1 Department of Orthodontics, Johann-Wolfgang Goethe University, Frankfurt, Theodor-Stern-Kai 7, 60596 Frankfurt, Germany; eslamishahrbabaki@med.uni-frankfurt.de (S.E.); sayahpour@med.uni-frankfurt.de (B.S.); plein@med.uni-frankfurt.de (N.P.); kopp@med.uni-frankfurt.de (S.K.); 2 Dental Practice, Frankfurter Street 3, 64293 Darmstadt, Germany; helena.m.selge@gmail.com; 3 Clinic for Oral, Maxillofacial, and Facial Surgery, University Hospital Basel, 4031 Basel, Switzerland; lukasbenedeikt.seifert@usb.ch; 4 Clinic for Maxillofacial and Plastic Surgery, Johann-Wolfgang Goethe University, Theodor-Stern-Kai 7, 60596 Frankfurt, Germany; r.sader@em.uni-frankfurt.de

**Keywords:** cleft lip and palate, Hotz’s plate, alveolar molding appliance, presurgical infant orthopedics, prospective studies

## Abstract

**Background:** This study investigated the effects of preoperative maxillary plates on cleft width reduction in infants with unilateral cleft lip and palate and their control group. The study also aimed to compare the digital and manual methods in measurement of changes in maxillary segment positioning in sagittal and transverse dimensions using digital 3D models and conventional plaster casts. **Methods:** Twenty infants with unilateral cleft lip and palate and their control group of eleven infants with isolated cleft palate were enrolled in a prospective interventional study (2020 to 2024). Participants were treated with either active or passive maxillary plates. Sagittal, transversal and angular measurements were taken both manually as well as digitally at three time points: 24–48 h postnatal (T0), approximately at six months old (T1, immediately before surgery), and one year postoperatively (T2). **Results:** Significant reductions in cleft width were observed across all patients over the treatment period, regardless of the type of plate used (*p* < 0.001). The mean cleft width reduction was 5.050 mm. Infants treated with active plates had a larger reduction in cleft width than those with passive plates (*p* = 0.024), averaging 5.846 mm compared to 3.571 mm. Neither the side of the cleft nor the patient’s gender influenced the degree of cleft reduction (*p* = 0.884 and *p* = 0.245, respectively). The study found significant modifications in the maxilla’s transverse, sagittal, and angular dimensions (*p* < 0.001). When comparing sagittal growth, the study group differed from the control group (*p* = 0.004), with isolated cleft palate patients showing more substantial sagittal expansion. Additionally, the overall change in the ITT’ distance differed significantly between the study and control groups over time (*p* < 0.001). Cleft size at baseline did not affect the extent of changes within the cleft area. No significant discrepancies were found between digital and manual measurement methods, confirming the reliability of both. **Conclusions:** Active plates demonstrated greater efficacy in cleft reduction for wider and more divergent clefts, while passive plates were suitable for smaller clefts.

## 1. Introduction

Cleft lip and palate (CLP) is considered to be one of the most common craniofacial deformities of our time [1,2]. It is estimated that 1 in 500–700 children is affected worldwide [3]. Despite the high prevalence of CLP, there is still no global, standardized concept of treatment [4] and the timing and number of cleft palate repair surgeries vary.

One-stage surgical cleft closure has been advocated to reduce the number of operations in these children [1]. Based on this treatment concept, the surgical closure of the lip, alveolar bone and soft palate takes place in one single operation at the age of approximately six months old in order to restore the anatomical structures early in life and simulate the growth of the maxilla [1,5]. This single surgery also encourages normal speech development and minimizes psychological trauma [1,6]. Furthermore, the early surgical procedure in the first year of life promotes healing with as little scarring as possible, as the child still has a partial embryonic ability to heal wounds [1].

Prior to surgical cleft repair, presurgical infant orthopedics (PIO) is performed using a maxillary plate [7], covering alveolar segments and the cleft area [8]. Due to the challenges that these patients face regarding food intake and speech development [9], the PIO using maxillary plates should be initiated as early as possible. These plates serve to separate the oral and nasal cavities and therefore act as an artificial palatal roof, which also facilitates food intake [10]. They help position the tongue in a more physiological anterior–caudal position, thereby stimulating the anterior development of the maxilla while preventing the tongue from interfering between the maxillary segments adjacent to the cleft [11,12,13]. In this way, they promote harmonious maxillary growth [14,15] and reduce the cleft width [16]. By bringing the cleft margins closer together, less tissue needs to be detached during surgery to close the cleft. This allows for a gentler and less traumatic operation [11].

These orthopedic maxillary plates are classified into two main types: active and passive appliances. Active plates (APs) are designed with screws to move the maxillary segments. The primary goal of this active movement is to harmonize the jaw segments and bring them closer together, ultimately reducing the width of the cleft. This is achieved by rotating and pivoting the segments rather than merely approximating them. Additionally, this mechanism encourages bone apposition [7].

In contrast, passive plates (PPs) do not incorporate active elements for narrowing the cleft. Instead, they focus on reducing the size of the cleft and promoting growth within the affected area through targeted block-outs on the maxillary model and the inner surface of the appliance.

The decision between using an active or passive plate for treatment primarily hinges on the initial size of the cleft and the alignment of the segments relative to each other. APs are typically favored for patients with larger cleft sizes, where there is a greater distance between the maxillary segments. As the APs require a sufficient alveolar ridge height to be effective, they are only used selectively.

Despite the purported benefits of this treatment approach, the evidence regarding the effects of these appliances remains controversial [17]. Various studies have already dealt with the morphological changes of the maxillary segments in CLP patients during PIO therapy, based on the measurement of digitized models using individual model analysis protocols [18,19,20]. Since the various concepts in the treatment of patients with cleft lip and palate differ between the specialized centers in terms of the number and timing of surgical cleft closure, the measurement times of the maxillary models vary greatly in scientific research. In addition, the majority of studies only use passive devices.

Therefore, this study aimed to assess the efficacy of AP and PP within the framework of the single-operation technique and ascertain whether there is a significant reduction in cleft size and treatment outcomes attributable to this therapy. Additionally, the study aimed to assess the comparative effectiveness of digital and manual measurement in assessing preoperative alveolar molding therapy in patients with UCLP.

## 2. Materials and Methods

### 2.1. Study Design and Ethics

This present monocentric prospective study received approval from the ethics committee of J. W. Goethe University Frankfurt under identifier 1540. Informed consent was obtained from the parents and legal guardians of all patients.

### 2.2. Patients and Study Groups

The CLP group consisted of 20 patients (16 males, 4 females; 11 left-sided and 9 right-sided clefts) and included two subgroups: group CLP-AP with treatment using active plates (14 patients) and group CLP-PP consisting of patients in treatment with passive plates (6 patients). The allocation of the patients in CLP-AP and CLP-PP groups was performed based on the patients’ individual anatomy, cleft size and orientation of the maxillary segments. A total of 11 patients with isolated cleft palate (4 females and 7 males) were assigned to the control group (group CP).

### 2.3. Inclusion and Exclusion Criteria

Only infants admitted to the cleft treatment center at J. W. Goethe University Frankfurt immediately after birth were recruited for this study. The CLP group (infants with cleft lip and palate) included newborn infants with nonsyndromatic, unoperated, complete one-sided cleft lip, alveolus and palate, whereas the CP group (control group) consisted of newborns with isolated cleft palate. Infants with systemic diseases, syndromes or other deformities or those who experienced delayed admission to the cleft center were excluded from the study. Additionally, only patients with clearly identifiable landmarks on their maxillary models were considered for inclusion in this study.

### 2.4. Sample Size Calculation

The recruitment took place between 2020 and 2024 until the required sample size was achieved.

The power calculation was based on a study by Sabri [21] in collaboration with the Institute for Biostatistics and Mathematical Modeling of J. W. Goethe University Frankfurt. In order to achieve a power of 90% at a significance level of 0.05, 20 patients were deemed necessary to detect a clinically relevant difference of 2 mm in cleft width between T0 (pretreatment) and T1 (post-PIO therapy).

### 2.5. Fabrication of Maxillary Plates

Impressions were taken from the patients’ maxilla 24–48 h after birth (T0), immediately preoperation, approximately at 6 months of age (T1), and one year postoperation, approximately 18 months of age (T2), using impression trays made from light-cured tray material (Triad VLC tray material, Dentsply Sirona, Charlotte, NC, USA) and alginate (Tetrachrom Alginat, Kaniedenta, Berlin, Germany). Afterwards, maxillary plaster models were made using type III hard stone (Sheraplaster, SHERA technology, Lemförde, Germany). The type of the plate (passive vs. active) was then decided based on the cleft size, alveolar ridge height and orientation of the maxillary segments. Following the block out of the cleft segment on the plaster model, both active plates (APs) and passive plates (PPs) were fabricated through thermoforming of a 1.5 mm thick multilayered thermoplastic foil (Durasoft, SCHEU-DENTAL, Iserlohn, Germany).

**Figure 1:**
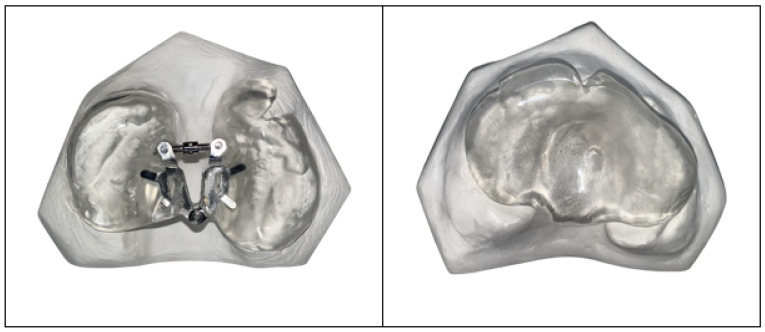
Representative image of plaster models with an active (**left**) and passive (**right**) plate.

The PP covered both jaw segments and the cleft area, whereas the AP consisted of two separate segments, each covering one-half of the alveolar ridge and connected by an open screw (Forestadent Bernhard Förster, Pforzheim, Germany) (Figure 1).

### 2.6. Presurgical Infant Orthopedic Therapy with Maxillary Plates

Parents were instructed that the plates should be worn 24 h a day and only be removed for daily cleaning. Additionally, parents of children in the CLP-AP group were directed to rotate the screw in the active plate once every three days, with each turn equivalent to 0.225 mm. Patients underwent monthly monitoring, during which the fit of the plates was assessed at each appointment. If any lack of fit was detected with the maxillary plate, a new plate of the same type was provided for the patient.

Six months into the presurgical infant orthopedic therapy and immediately prior to surgery (T1), as well as approximately one year after surgical cleft closure (T2), new impressions were taken from the patients’ maxilla.

### 2.7. Measurements

The models were analyzed using two methods: manual and digital measurements. Manual measurements were conducted using calipers (smiledental, Düsseldorf, Germany), while angles were measured with a geometry triangle and a transparent ruler with 0.01 mm accuracy. Following manual measurements, the models were digitized using the Edge 3D model scanner (DOF Europe GmbH, Wiesbaden, Germany) and the measurements were then performed on the digital data set using the OnyxCeph^3TM^ software (Version 3.2, Image Instruments, Chemnitz, Germany).

**Figure 2:**
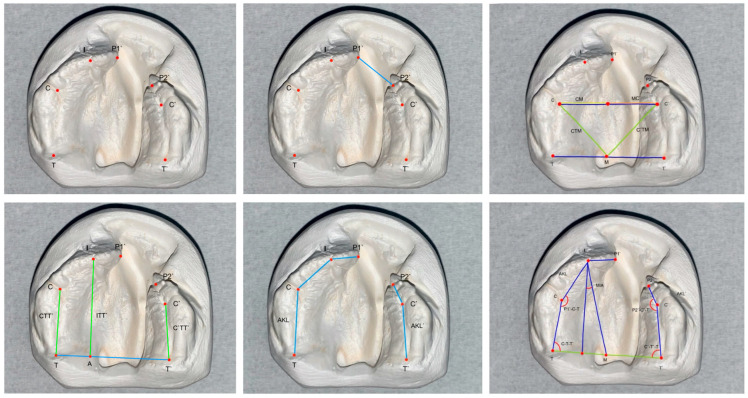
Representative image of digital models with markings P1’ at the anterior edge of the long segment and P2’ on the anterior edge of the short segment of the unilateral cleft located on the left side.

The measurements were performed before (T0) and after PIO treatment (T1) as well as approximately one year after surgical cleft closure (T2), evaluating cleft width, transverse, sagittal and angular parameters. The landmarks used in the present study were selected based on previous studies by Mazaheri et al. [22], Ashley-Montague [23], Sillman [24], García et al. [25] and Burgaz [26] (Table 1, Figure 2).

### 2.8. Statistical Analysis

The statistical evaluation was carried out under the supervision of J. W. Goethe University.

Statistical analyses and graphical representation were performed with JASP, version 0.19.3 (The JASP Team, The Netherlands).

The normality of the data distribution was evaluated through the Shapiro–Wilk test. In order to compare the effects of active plates and passive plates (group CLP-AP vs. group CLP-PP), the role of gender (male vs. female) and the side of cleft (right side vs. left side), a *t*-test for independent samples was performed, while non-normally distributed values were analyzed using the Wilcoxon Mann–Whitney test.

For normally distributed dependent data, a paired *t*-test was applied for T0–T1, T0–T2 and T1–T2 comparisons. For non-normally distributed data, a Wilcoxon signed-rank test was applied.

The intercorrelation coefficient (ICC) was calculated to assess differences between manually and digitally determined values. ICC values range from −1 to 1: 

ICC = 1: Perfect agreement;

ICC > 0.9: Excellent reliability;

0.75 < ICC ≤ 0.9: Good reliability;

0.5 < ICC ≤ 0.75: Moderate reliability;

ICC < 0.5: Poor reliability.

A confidence interval of 95% was used to determine significance; if the interval did not include 0, the results were considered significant. Interpretation of reliability followed the criteria of Koo et al. [27]. To evaluate the agreement between the manual and digital measuring methods, a Bland–Altman analysis was performed.

All measurements were performed by the same investigator. Due to the possible measurement errors, randomly selected models were measured a second time after at least four weeks and the individual measurement error was calculated using the formula according to Dahlberg. Using this analysis, an individual method error of less than 0.5 mm was determined, which was in the acceptable range determined by Seckel et al. [28].

The level of significance (α-error) was set at 5%. A *p*-value of less than 0.05 was deemed to indicate statistical significance for all statistical analyses.

## 3. Results

Since the cleft-related measurements could only be performed in the presence of an alveolus cleft, they could not be performed in the control group with isolated palatal cleft at any time point.

Since the surgical closure of the cleft at T2 also eliminated the clefts in both CLP-AP and CLP-PP groups, these measurements were only performed at T0 and T1 in these groups.

### 3.1. Cleft Width

**Figure 3:**
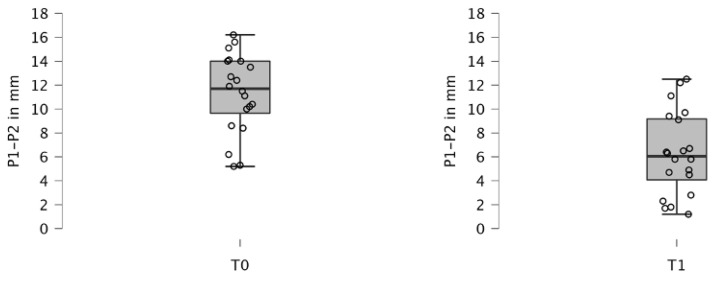
Boxplot of cleft width (P1–P2 measurement) before (T0) and after treatment (T1) in the overall study population.

**Figure 4:**
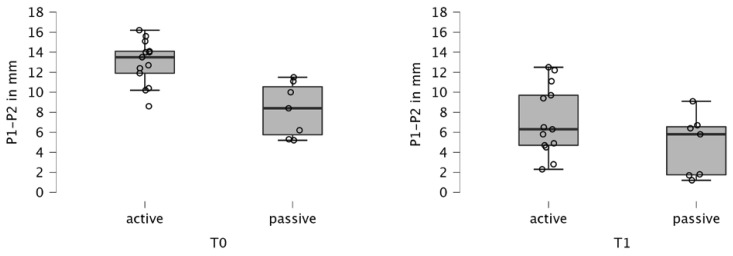
Boxplot of cleft width (P1–P2 measurement) before (T0) and after treatment (T1), stratified according to the type of the plate (passive vs. active).

PIO using maxillary plates significantly reduced cleft width in all 20 patients (*p* < 0.001) (Table 2, Figure 3 and Figure 4). Prior to treatment (T0), the cleft width averaged 11.320 (±3.296) mm, which decreased to 6.270 (±3.475) mm after 6 months of therapy (T1), indicating an approximate reduction of 5 mm in cleft size (*p* < 0.001).

Intergroup comparisons revealed a significantly greater reduction in cleft width in CLP-AP (*p* = 0.024). It is important to note that patients with larger cleft widths were assigned to group CLP-AP, potentially introducing a bias favoring this group in terms of efficacy in cleft width reduction. To address this potential bias, a multivariate linear regression analysis was conducted to examine the influence of group allocation on the change in millimeters while controlling for cleft width at time T0. Results indicate that even after adjusting for the effect of cleft width at time T0, a significant difference in outcomes between the plate types in favor of the active plate remains apparent (*p* = 0.040).

No statistically significant differences were found regarding patients’ gender or the cleft side (*p* = 0.245 and 0.884, respectively) (Table 2).

### 3.2. Transversal Measurements

No significant changes in the anterior arch width (CC’) were observed in CLP-AP or CLP-PP groups (Table 3). On the other hand, the control group (CP) showed a significant increase in the anterior arch width between T1–T2 and T0–T2 (*p* < 0.001).

The posterior alveolar arch width (TT’) did not increase significantly during T0–T1. In the CLP-AP and CP groups, TT’ increased between T1–T2 (*p* = 0.04; *p* < 0.001) and T0–T2 (*p* < 0.001) significantly.

The distance CTM of the large segment increased over all observation periods T0–T1 (*p* = 0.04; *p* = 0.05), T1–T2 (*p* = 0.02; *p* < 0.001) and T0–T2 (*p* < 0.001) in the CLP-AP and CP groups and between T0–T1 (*p* = 0.04) and T0–T2 (*p* < 0.001) in the CLP-PP group. The distance C’T’M of the small segment enlarged in CLP-AP between T1–T2 and T0–T2 (*p* < 0.001), in CLP-PP between T0 and T2 (*p* = 0.02) and in CP in all time periods (*p* < 0.001).

### 3.3. Sagittal Measurements

No significant changes were observed in the CLP-AP or CLP-PP groups regarding the distance P1/P1’-TT’ of the large segment over T0–T1 (Table 4). In CLP-AP, a significant increase in the distance P2/P2’-TT’ of the small segment was found (*p* < 0.001). The overall sagittal distance I-TT’ increased significantly in CLP-AP (T0–T1 and T0–T2; *p* < 0.001), in CLP-PP (T0–T2; *p* < 0.001) and CP (T0–T1, T1–T2, T0–T2; *p* < 0.001). While the distance I-CC’ increased within T0–T1 in all groups, it decreased in all groups after the surgical cleft closure. This change was significant for CLP-PP (*p* = 0.03) and CP (*p* < 0.001). In CLP-AP, distances of C-TT’ (T1–T2, T0–T2; *p* < 0.001) and C’-TT’ (T0–T1 *p* < 0.001; T1–T2, *p* = 0.01; T0–T2, *p* < 0.01) increased over the observation period. CLP-PP patients showed a significant increase for C’-TT’ postsurgery (T1–T2; *p* < 0.001). In the CP group, C-TT’ and C’-TT’ increased in all time periods (*p* < 0.001).

### 3.4. Segmental Arch Measurements

Mesial alveolar ridge length P1/P1’-I of the large segment and P2/P2’-I of the small segment increased in both CLP groups (CLP-AP *p* < 0.01; *p* < 0.001 and CLP-PP *p* = 0.03; *p* = 0.01) within T0–T1 (Table 5). The medial alveolar ridge length of the large segment I-C increased in CLP-AP between T1–T2 and T0–T2 (*p* < 0.001). In CLP-PP, I-C increased between T0–T1 (*p* = 0.02) and T0–T2 (*p* < 0.001). For the distal alveolar ridge length of the large segment C-T, there could be shown to be an increase for all groups (CLP-AP: T1–T2, T0–T2 *p* < 0.001; CLP-PP: T1–T2 *p* = 0.04; CP: T0–T1, T1–T2, T0–T2 *p* < 0.01). The small segment’s distal alveolar ridge length (C’-T’) also enlarged in all groups (CLP-AP: T0–T1, T1–T2, T0–T2 *p* < 0.001; CLP-PP: T1–T2 *p* < 0.001; CP: T0–T1 *p* = 0.01 T1–T2, T0–T2 *p* < 0.001). The total alveolar ridge length of the large segment (AKL) changed in both CLP groups, while only in the CLP-AP group was the change significant (T0–T1 *p* < 0.001; T0–T2 *p* = 0.01). The enlargement of the total alveolar ridge length of the small segment (AKL’) increased in the CLP-AP (T1–T2 *p* = 0.02, T0–T2 *p* < 0.001) and CLP-PP (T0–T1 *p* = 0.03, T0–T2 *p* < 0.01) groups significantly.

### 3.5. Angular Measurements

The midline deviation of point I reduced significantly in the CLP-AP group during the preoperative orthodontic treatment T0–T1 (*p* < 0.001) (Table 6). Both appliance types could reduce the midline deviation of point Inzisale within T1–T2 (*p* < 0.001; *p* = 0.02) and T0–T2 (*p* < 0.01; *p* = 0.03). Throughout the observation period, the curvature of the distal alveolar ridge in the large segment remained unchanged, regardless of the plate type. In contrast, the curvature of the distal alveolar ridge in the small segment showed a significant reduction in CLP-PP within T0–T1.

### 3.6. Manual Versus Digital Measurement

The analysis of digital and manual measurement results reveals a high level of agreement between the two methods (Table 7). Out of a total of 96 measurements, 79 (82.29%) show an ICC value greater than 0.75, indicating high reliability. Furthermore, 62 values (64.58%) have an ICC value above 0.9, suggesting excellent reliability.

## 4. Discussion

The primary objective of the present study was to investigate the efficacy of treatment with maxillary plates prior to early surgical cleft closure, adhering to the one-phase surgical closure treatment concept, in reducing the cleft width among patients diagnosed with unilateral cleft lip, alveolus, and palate (UCLP). The findings of this study indicate that a significant reduction in cleft size can be achieved within a relatively short period of 6 months through infant orthopedic therapy, utilizing both passive and active plates. This reduction facilitates early surgical cleft closure, underscoring the effectiveness of the treatment approach in addressing unilateral UCLP.

Despite the controversial nature of evidence concerning the role of infant orthopedic therapy in patients with UCLP, our study findings are congruent with those of comparable studies [25,29,30,31,32]. The demographic characteristics of our study group were also similar to those of comparable studies [25,33,34,35], with a prevalence of male infants born with a unilateral cleft and a tendency towards the left side.

A crucial controversy revolves around the utilization of active plates in the treatment of patients with UCLP [17]. While active plates receive criticism regarding their unwanted maxillary compression, their function is to change the orientation of the segments and bring them together without compression of the maxilla. It should also be noted that the primary therapeutic goal is to improve the initial surgical situation to enable the smoothest and most atraumatic operation possible. In the later course of growth, bone should form in the cleft area [17].

Compared to the active plate, the passive plate works by empirically blocking out, and thus can positively promote growth toward the cleft areas. In our study, active plates demonstrated a greater reduction in cleft width compared to passive plates, indicating their efficacy, particularly in patients with larger cleft widths. Although a 2.4 mm difference might seem small, it is clinically significant when considered in relation to the total cleft width (12.97 mm at T0 in the AP group, accounting for 20% of the initial width, and 7.131 mm at T1, accounting for 33% of the pre-operative width). This 20–30% reduction is considered clinically significant and provides two key advantages for clinicians. Firstly, a 2.4 mm reduction through the use of active plates can allow the subsequent use of passive plates without the risk of trauma or injury due to poor fit. Secondly, it increases the success of one-stage surgical repair by minimizing cleft size, thereby reducing issues such as high tension on the soft tissue.

Assessment of the anterior alveolar arch width showed that there is no compression of the dental arch in the preoperative treatment period, neither in the CLP-AP group treated with active plates nor in the CLP-PP group treated with passive appliances or in the control cleft palate group also treated with passive plates. In the postoperative period, only in the control group of cleft palate patients was there a significant increase compared to the initial situation, which may be due to the reduced cleft formation, as the lip and alveolar ridge are not affected by the cleft formation. Therefore, there is a different, less developed postoperative scar tissue than in the CLP group, which counteracts the growth in width. Similar results were also described by Neuschulz et al. [36], Braumann et al. [37] and Hoffmannova et al. [38], who found that in patients with complete CLP treated with passive plates, the anterior arch width remained largely constant within a treatment period of 12 months (presurgical). In contrast, an evaluation by Ambrosio et al. [39] showed a significant reduction for the anterior arch width C-C’ before and after surgical intervention when preoperative orthopedic treatment was not part of the treatment protocol. Moreover, our investigation demonstrated that both active and passive plates supported continued transverse growth and prevented compression in the tuberosity region (TT’) within the preoperative treatment period T0–T1. Postsurgery, the posterior arch width increased significantly in the CLP-AP and CP groups. Scientific research by Neuschulz et al. [36], Braumann et al. [37], Hoffmannova et al. [38] and Ambrosio et al. [39] also showed a posterior width increase after 12 months. The changes in distances CTM (large segment) and C’T’M (small segment) during the treatment period indicate a combination of transversal and sagittal growth of the alveolar segments. However, this growth is not analyzed in isolation, as it may involve multiple directional changes.

The study demonstrated that the cleft pole of the large segment maintains a stable sagittal position in the CLP-AP and CLP-PP groups, while the cleft pole of the small segment moves progressively further in the CLP-AP patients. This reflects the therapeutic effects of active plates. In cases of CLP, the small jaw segment is often positioned further dorsally and is encouraged to develop ventrally through active plate therapy, facilitating its approximation to the large segment. Additionally, the therapy aims to promote the growth of the large segment toward the cleft area and support a shift of the centerline toward the cleft side. Therefore, active plates are especially used in cases with segment discrepancies in thesagittal direction.

While the overall sagittal distance I-TT’ over the whole treatment period T0–T2 increased significantly in all groups, there could be shown to be differences in the anterior and posterior sagittal part of the jaw segments. After surgical cleft closure, the distance from point I to CC’ decreases, while the distance of the C-point between the large and small segments increases. This is most likely due to scar tension, which appears to be stronger in the anterior region than in the posterior region. Our results are in agreement with other current scientific research. A study by Braumann et al. [37] also demonstrated that the anterior alveolar arch length increased during orthodontic PSIO therapy with passive plates before surgical intervention and decreased after cleft lip closure. The posterior alveolar arch length increased pre- and postoperatively, comparable to our results. In a scientific work by Neuschulz et al. [36], the anterior alveolar arch length during PSIO therapy remained largely constant, while the posterior length increased before and after surgical intervention. In contrast to our results, neither the sagittal distance I-TT’ nor the posterior alveolar arch length during preoperative intervention changed significantly in a study by Hoffmannova et al. [38]. In accordance with our results, Ambrosio et al. [19] showed that the I-C/C’ distance decreases after surgical interventions, while the distance I-T/T’ increased.

In contrast to our results, Braumann et al. [37] demonstrated that the alveolar crest length AKL of the large segment and AKL’ of the small segment increased both on the cleft side and on the non-cleft side in CLP-PP patients within the first year of life. In accordance with our study, Neuschulz et al. [36] showed that the segmental arch length in patients treated with passive plates did not change significantly on the non-cleft side (large segment), while it increased significantly on the cleft side.

This study could show that the midline deviation could be significantly reduced in the CLP-AP group during the preoperative orthodontic treatment T0–T1, and both appliance types (CLP-AP and CLP-PP) could reduce the midline deviation over the whole treatment period. In the CLP-PP group undergoing orthodontic preoperative therapy, the significant reduction in the curvature of the distal alveolar ridge in the small segment supports the recognition of the growth-directing effect of passive plates.

The general comparability of study results is limited, as different studies use varying evaluation protocols and lack a standardized nomenclature. Additionally, the literature primarily focuses on the effects of passive appliances, making comparisons for active plates more challenging. The majority of previous studies on active plates examine their long-term effects in children and adolescents [40,41,42], focus on fixed appliances like the Latham appliance [42,43,44,45] or deal with the therapy of bilateral cleft lip and palate [46,47].

The analysis of digital and manual measurement results reveals a high level of agreement between the two methods. However, for the total segmental arch lengths (AKL and AKL’), ICC values below 0.5 appear more frequently. This may indicate an accumulation of measurement errors, as these values represent summed measurement distances. In accordance with our findings, a study by Budak et al. [48] found a high intraclass correlation greater than 0.90 for several extraoral measurement points of patients with unilateral and bilateral cleft lip and palate in the digital analysis when compared to the manual method. A systematic review by Luu et al. [49] concluded that virtual study models are clinically acceptable compared with plaster study models with regard to intrarater reliability and validity of selected linear measurements. Luu et al. highlighted that a clinically significant threshold should be set at 0.5 mm. In the present study, the average values across all measurement methods were closely aligned. Our study demonstrated that digital measurements of digitized plaster models are just as reliable as manual measurements of physical plaster models. This supports the idea that, with the advancements in digitalization, digital impressions combined with digital model measurements can yield equally accurate results comparable to previous study findings. Furthermore, thanks to digital progress, additional and expanded measurement methods are conceivable for future studies. These advancements could enable precise and risk-free digital assessments of various therapeutic effects.

### Limitations

The demographics of the models as well as the participants were quite narrow in this study, which affects the generalizability of the results adversely. Data were analyzed per protocol, which always carries a risk for bias.

Another limitation of the present study was the lack of numerical or categorical guidelines for the allocation of patients into the active or passive plate groups. Further studies comparing both methods are indeed required to establish such values, which can later serve as guidelines for patient allocation.

## 5. Conclusions

Under the limitations of our study, it can be concluded that infant orthopedic therapy utilizing passive or active maxillary plates effectively reduces cleft size prior to early surgical cleft closure. Active plates demonstrate superior efficacy in reducing cleft width compared to passive plates and can be recommended in patients with wider clefts. Additionally, both manual and digital measurements of plaster models demonstrate equal accuracy.

## Figures and Tables

**Table 1:** Definition of the landmarks and the linear measurements.

Abbreviation	Landmark	Definition
I	Incisal Point	Intersection point of the alveolar ridge with the connecting line of the incisive papilla and the labial frenulum
P1	Alveolar Gap Point Long	Outermost and foremost point of the alveolar ridge, long segment
P2	Alveolar Gap Point Short	Outermost and foremost point of the alveolar ridge, short segment
C	C-Point Right	Lateral Sulcus Point Intersection point between the lateral sulcus and the alveolar ridge line on the right side
C’	C-Point Left	Lateral Sulcus Point Intersection point between the lateral sulcus and the alveolar ridge line on the left side
T	Tubercle Point Right	Most distal point of the alveolar ridge on the right side
T’	Tubercle Point Left	Most distal point of the alveolar ridge on the left side
M	Midpoint	Midpoint of the line segment TT’
A	A-Point	Intersection point of the perpendicular from the TT’ line through point I
CT	CT-Point	Intersection point of the perpendicular from the TT’ line through point C
CIA	CIA-Point	Intersection point between CC’ line and IA line

**Table 2:** Descriptive analysis as well as comparisons of cleft width in CLP group at T0 (pretreatment), T1 (6 months into the therapy and immediately prior to the operation) and T2 (1 year following the operation) both overall and stratified according to type of appliance (passive vs. active), gender (male vs. female) and cleft side (right vs. left). N = sample size, SD = standard deviation, PP = passive plate, AP = active plate, M = male, F = female, L = left side, R = right side.

Type of Stratification		Measurement Time	N	Mean (mm)	SD (mm)	*p*-Value
Overall assessment		T0	20	11.320	3.296	NA
		T1	20	6.270	3.475	NA
		T0–T1	20	5.050	2.210	<0.001 *^A^
Type of appliance	AP	T0	14	12.9777	2.245	NA
		T1	14	7.131	3.474	NA
		T0–T1	14	5.846	2.334	<0.001 *^A^ 0.024 **^B^
	PP	T0	6	8.243	2.707	NA
		T1	6	4.671	3.085	NA
		T0–T1	6	3.571	0.804	<0.001 *^A^ 0.024 **^B^
Gender	M	T0	16	11.313	3.577	NA
		T1	16	5.969	3.690	NA
		T0–T1	16	5.344	2.264	<0.001 *^A^ 0.245 ^C^
	F	T0	4	11.350	2.198	NA
		T1	4	7.475	2.445	NA
		T0–T1	4	3.875	1.735	<0.001 *^A^ 0.245 ^C^
Cleft side	L	T0	11	10.345	3.707	NA
		T1	11	5.227	3.449	NA
		T0–T1	11	5.118	1.847	<0.001 *^A^ 0.884 ^D^
	R	T0	9	12.511	2.392	NA
		T1	9	7.544	3.238	NA
		T0–T1	9	4.967	2.706	<0.001 *^A^ 0.884 ^D^

* Statistical significance of dependent samples *t*-test (*p* < 0.05), ** statistical significance of independent samples *t*-test for (*p* < 0.05), A: T0–T1 intragroup comparisons within each subcategory, B: AP vs. PP intergroup comparisons, C: M vs. F intergroup comparisons, D: L vs. R intergroup comparisons, NA = not applicable.

**Table 3:** Descriptive analysis as well as comparisons of the transversal measurements in CLP patients (groups CLP-AP and CLP-PP) and their control group (CP) at T0 (pretreatment), T1 (6 months into the therapy and immediately prior to the operation) and T2 (1 year following the operation). SD = standard deviation, IQR = interquartile range, PP = passive plate, AP = active plate, CC’ = anterior alveolar arch width, TT’ = posterior alveolar arch width, CTM = distance from right C-point to the midpoint of the line CC’, C’TM = distance from left C-point to the midpoint of the line CC’, CM = distance from right C-point to the midpoint of the line TT’, MC’ = distance from left C-point to the midpoint of the line TT’, AM = midline deviation of the incisal point.

Group	Time	CC’	TT’	CTM	C’T’M	CM	MC’	AM
		Mean/Median ± SD/IQR (mm)	Mean/Median ± SD/IQR (mm)	Mean/Median ± SD/IQR (mm)	Mean/Median ± SD/IQR (mm)	Mean/Median ± SD/IQR (mm)	Mean/Median ± SD/IQR (mm)	Mean/Median ± SD/IQR (mm)
CLP-AP	T0	26.10 ± 2.52	30.51 ± 2.40	19.08 ± 2.57	18.64 ± 1.97	12.92 ± 1.88	13.12 ± 1.99	0.74 ± 7.89
	T1	27.61 ± 4.19	31.42 ± 2.47	21.45 ± 3.45	19.77 ± 1.89	14.450 ± 2.75	13.07 ± 2.33	0.60 ± 6.94
	T2	27.09 ± 3.07	34.21 ± 3.33	23.45 ± 3.11	22.81 ± 2.45	13.49 ± 1.43	13.46 ± 2.91	0.49 ± 3.05
	T0–T1	1.51 ± 5.51	0.91 ± 2.57	2.37 ± 3.95 *^A^	1.13 ± 2.09	1.00 ± 2.77	−0.05 ± 2.42	−0.14 ± 5.78
	T1–T2	−0.52 ± 5.08	2.79 ± 4.61 *^B^	2.00 ± 2.71 *^B^	3.04 ± 3.18 *^B^	−7.96 ± 3.69	0.39 ± 3.18	−0.11 ± 5.69
	T0–T2	0.99 ± 3.93	3.90 ± 3.99 *^C^	4.37 ± 3.39 *^C^	4.17 ± 3.77 *^C^	0.57 ± 2.57	0.34 ± 3.08	−1.90 ± 12.93
CLP-PP	T0	24.62 ± 2.84	32.58 ± 4.49	18.4 ± 2.67	19.47 ± 1.93	11.73 ± 1.41	12.83 ± 1.85	−1.455 ± 5.28
	T1	25.82 ± 2.61	33.22 ± 4.25	20.38 ± 2.10	20.72 ± 1.73	12.50 ± 1.97	13.27 ± 1.47	−0.12 ± 9.69
	T2	25.55 ± 4.39	35.65 ± 3.54	24.55 ± 4.35	24.42 ± 2.72	12.07 ± 2.40	13.30 ± 2.88	−1.30 ± 2.56
	T0–T1	1.20 ± 2.84	0.63 ± 1.71	1.98 ± 1.76 *^A^	1.25 ± 1.25	0.77 ± 2.72	0.43 ± 1.30	1.33 ± 7.34
	T1–T2	−0.27 ± 4.04	2.43 ± 2.97	4.17 ± 4.55	3.70 ± 3.48	−0.43 ± 3.16	0.03 ± 1.73	−1.18 ± 11.18
	T0–T2	0.93 ± 1.96	3.07 ± 3.26	6.15 ± 3.67 *^C^	4.95 ± 3.63 *^C^	0.33 ± 1.99	0.47 ± 1.88	0.15 ± 6.62
CP	T0	20.18 ± 2.24	27.78 ± 3.86	17.13 ± 2.50	17.18 ± 2.08	NA	NA	NA
	T1	21.75 ± 3.62	27.91 ± 3.01	20.79 ± 3.57	20.63 ± 2.91	NA	NA	NA
	T2	25.95 ± 2.39	33.45 ± 2.65	25.26 ± 2.07	25.354	NA	NA	NA
	T0–T1	1.57 ± 2.46	0.13 ± 1.88	3.66 ± 2.86 *^A^	3.45 ± 2.61 *^A^	NA	NA	NA
	T1–T2	4.19 ± 1.96 *^B^	5.55 ± 3.62 *^B^	4.47 ± 2.61 *^B^	4.73 ± 2.36 *^B^	NA	NA	NA
	T0–T2	5.76 ± 2.02 *^C^	5.67 ± 4.07 *^C^	8.14 ± 2.19 *^C^	8.17 ± 1.62 *^C^	NA	NA	NA

* Statistical significance of paired sample *t*-test (*p* < 0.05), A: T0–T1 intragroup comparisons within each subcategory, B: T1–T2 comparisons, C: T0–T2 comparisons, NA = not applicable.

**Table 4:** Descriptive analysis as well as comparisons of the sagittal measurements in CLP patients (groups CLP-AP and CLP-PP) and their control group (CP) at T0 (pretreatment), T1 (6 months into the therapy and immediately prior to the operation) and T2 (1 year following the operation). SD = standard deviation, IQR = interquartile range, PP = passive plate, AP = active plate.

Group	Time	P1/P1‘-TT‘	P2/P2‘-TT’	I-TT’ (=I-A)	I-CC’	C-TT’	C‘-TT’
		Mean/Median ± SD/IQR (mm)	Mean/Median ± SD/IQR (mm)	Mean/Median ± SD/IQR (mm)	Mean/Median ± SD/IQR (mm)	Mean/Median ± SD/IQR (mm)	Mean/Median ± SD/IQR (mm)
CLP-AP	T0	23.72 ± 3.31	18.27 ± 2.20	22.81 ± 2.62	8.66 ± 2.01	13.83 ± 3.31	13.21 ± 1.84
	T1	24.83 ± 3.60	21.58 ± 2.31	25.24 ± 2.84	9.35 ± 3.13	15.69 ± 2.57	15.58 ± 1.37
	T2	NA	NA	26.48 ± 3.47	7.58 ± 3.17	19.73 ± 4.56	19.07 ± 4.56
	T0–T1	1.11 ± 3.40	3.31 ± 2.66 *^A^	1.90 ± 1.70 **^A^	1.40 ± 1.17	1.10 ± 2.35	2.36 ± 2.55 *^A^
	T1–T2	NA	NA	1.24 ± 3.88	−1.77 ± 3.10	4.04 ± 3.61 *^B^	3.49 ± 4.37 *^B^
	T0–T2	NA	NA	3.67 ± 3.15 *^C^	−1.08 ± 3.79	5.90 ± 5.49 *^B^	5.86 ± 5.19 *^C^
CLP-PP	T0	22.20 ± 1.87	18.92 ± 1.73	20.98 ± 3.24	6.53 ± 1.80	14.07 ± 2.62	14.62 ± 2.04
	T1	25.67 ± 3.69	22.28 ± 2.20	26.78 ± 4.32	11.42 ± 2.43	16.20 ± 3.78	13.93 ± 2.21
	T2	NA	NA	25.97 ± 2.55	7.52 ± 1.35	19.52 ± 3.74	17.83 ± 2.20
	T0–T1	3.47 ± 5.34	3.37 ± 3.84	5.80 ± 7.37	4.88 ± 3.88 *^A^	2.13 ± 6.36	−0.72 ± 3.76
	T1–T2	NA	NA	−0.82 ± 5.27	−3.90 ± 3.15 *^B^	3.32 ± 3.11	4.20 ± 1.54 *^B^
	T0–T2	NA	NA	4.98 ± 2.94 *^C^	0.98 ± 1.99	5.45 ± 5.84	3.48 ± 3.37
CP	T0	NA	NA	20.00 ± 2.72	6.11 ± 1.53	13.65 ± 2.17	14.09 ± 2.55
	T1	NA	NA	24.05 ± 2.59	14.63 ± 6.03	17.56 ± 3.18	18.01 ± 3.07
	T2	NA	NA	28.88 ± 3.46	7.35 ± 2.27	21.59 ± 2.29	21.85 ± 2.15
	T0–T1	NA	NA	4.05 ± 2.94 *^A^	8.52 ± 5.49 *^A^	3.92 ± 3.05 *^A^	3.92 ± 3.55 *^A^
	T1–T2	NA	NA	4.84 ± 3.64 *^B^	−7.27 ± 5.10 *^B^	4.03 ± 3.12 *^B^	3.84 ± 3.05 *^B^
	T0–T2	NA	NA	8.88 ± 3.37 *^C^	1.25 ± 2.43	7.95 ± 2.58 *^C^	7.75 ± 2.45 *^C^

* Statistical significance of paired sample *t*-test (*p* < 0.05), ** statistical significance of Wilcoxon test (*p* < 0.05), A: T0–T1 intragroup comparisons within each subcategory, B: T1–T2 comparisons, C: T0–T2 comparisons, NA = not applicable.

**Table 5:** Descriptive analysis as well as comparisons of the segmental arch measurements in CLP patients (groups CLP-AP and CLP-PP) and their control group (CP) at T0 (pretreatment), T1 (6 months into the therapy and immediately prior to the operation) and T2 (1 year following the operation). SD = standard deviation, IQR = interquartile range, PP = passive plate, AP = active plate.

Group	Time	P1/P1’-I	I-C	C-T	AKL (P1-I-C-T)	P2/P2’-C’	C‘-T‘	AKL’ (P2-C’-T’)
		Mean/Median ± SD/IQR (mm)	Mean/Median ± SD/IQR (mm)	Mean/Median ± SD/IQR (mm)	Mean/Median ± SD/IQR (mm)	Mean/Median ± SD/IQR (mm)	Mean/Median ± SD/IQR (mm)	Mean/Median ± SD/IQR (mm)
CLP-AP	T0	7.29 ± 2.03	10.64 ± 2.03	14.10 ± 3.58	28.53 ± 6.15	6.80 ± 1.40	13.59 ± 1.64	22.86 ± 5.35
	T1	8.89 ± 2.16	13.09 ± 4.09	16.00 ± 2.63	35.52 ± 7.21	8.98 ± 1.92	15.90 ± 1.41	25.47 ± 3.76
	T2	NA	14.97 ± 3.54	20.28 ± 4.52	35.09 ± 3.98	NA	19.64 ± 4.77	31.05 ± 6.39
	T0–T1	1.60 ± 2.28 *^A^	1.89 ± 4.37	0.80 ± 2.45	6.99 ± 4.91 *^A^	2.18 ± 2.23 *^A^	2.31 ± 2.20 *^A^	2.61 ± 5.81
	T1–T2	NA	1.89 ± 4.37 *^B^	4.28 ± 3.63 *^B^	−0.43 ± 9.45	NA	4.80 ± 4.27 **^B^	5.58 ± 7.60 *^B^
	T0–T2	NA	4.34 ± 3.93 *^C^	6.18 ± 5.95 *^C^	6.56 ± 8.53 *^C^	NA	6.75 ± 7.03 **^C^	8.19 ± 6.83 *^C^
CLP-PP	T0	6.62 ± 2.06	9.87 ± 2.77	14.88 ± 2.57	30.18 ± 4.51	7.23 ± 1.40	15.07 ± 1.99	22.22 ± 3.77
	T1	11.27 ± 2.36	14.53 ± 2.93	16.77 ± 3.69	32.18 ± 5.66	11.40 ± 1.53	14.35 ± 2.24	30.22 ± 6.37
	T2	NA	15.63 ± 2.46	20.15 ± 4.07	35.50 ± 3.94	NA	18.55 ± 2.19	31.40 ± 3.78
	T0–T1	3.15 ± 2.78 **^A^	4.67 ± 3.28 *^A^	1.88 ± 6.11	2.00 ± 6.85	4.17 ± 1.56 *^A^	−0.72 ± 3.76	6.65 ± 2.85 **^A^
	T1–T2	NA	1.10 ± 3.60	3.38 ± 3.07 *^B^	3.32 ± 7.74	NA	4.20 ±1.54 *^B^	1.18 ± 7.16
	T0–T2	NA	5.77 ± 3.02 *^C^	5.27 ± 6.21	5.32 ± 6.22	NA	3.48 ± 3.37	9.18 ± 2.48 *^C^
CP	T0	NA	NA	14.33 ± 1.84	NA	NA	14.75 ± 2.28	NA
	T1	NA	NA	18.00 ± 3.07	NA	NA	17.81 ± 3.12	NA
	T2	NA	NA	21.99 ± 2.28	NA	NA	22.20 ± 2.09	NA
	T0–T1	NA	NA	3.67 ± 2.85 *^A^	NA	NA	3.06 ± 3.39 *^A^	NA
	T1–T2	NA	NA	3.99 ± 3.18 *^B^	NA	NA	4.39 ± 3.78 *^B^	NA
	T0–T2	NA	NA	7.66 ± 2.52 *^C^	NA	NA	7.45 ± 2.54 *^C^	NA

* Statistical significance of paired sample *t*-test (*p* < 0.05), ** statistical significance of Wilcoxon test (*p* < 0.05), A: T0–T1 intragroup comparisons within each subcategory, B: T1–T2 comparisons, C: T0–T2 comparisons, NA = not applicable.

**Table 6:** Descriptive analysis as well as comparisons of the angle measurements in CLP patients (groups CLP-AP and CLP-PP) and their control group (CP) at T0 (pretreatment), T1 (6 months into the therapy and immediately prior to the operation) and T2 (1 year following the operation). SD = standard deviation, IQR = interquartile range, PP = passive plate, AP = active plate.

Group	Time	MIA	C-T-T’	P1/P1’-C-T	C’-T-T’	P2/P2’-C’-T’
		Mean/Median ± SD/IQR (°)	Mean/Median ± SD/IQR (°)	Mean/Median ± SD/IQR (°)	Mean/Median ± SD/IQR (°)	Mean/Median ± SD/IQR (°)
CLP-AP	T0	17.51 ± 5.43	80.99 ± 8.46	129.50 ± 10.24	79.61 ± 7.35	153.59 ± 11.60
	T1	12.59 ± 5.83	80.74 ± 8.28	124.61 ± 10.91	79.42 ± 5.17	151.21 ± 11.62
	T2	5.63 ± 4.37	77.91 ± 6.23	NA	79.86 ± 9.08	NA
	T0–T1	−4.93 ± 5.92 *^A^	−0.26 ± 9.85	−4.89 ± 15.90	−0.19 ± 9.53	−2.39 ± 17.00
	T1–T2	−6.96 ± 6.74 *^B^	−2.82 ± 9.52	NA	−0.65 ± 6.70	NA
	T0–T2	−11.89 ± 7.23 *^C^	−3.08 ± 9.58	NA	−2.10 ± 6.60	NA
CLP-PP	T0	10.50 ± 3.78	73.53 ± 8.32	132.55 ± 10.42	76.98 ± 5.16	147.08 ± 14.04
	T1	12.67 ± 5.40	75.98 ± 16.44	125.95 ± 24.54	76.80 ± 9.96	147.35 ± 17.29
	T2	5.62 ± 1.60	76.35 ± 9.02	NA	76.35 ± 9.02	NA
	T0–T1	2.17 ± 8.81	2.45 ± 22.12	−6.60 ± 24.07	−0.18 ± 14.18 *^A^	0.27 ± 9.90
	T1–T2	−7.05 ± 5.39 *^B^	0.65 ± 13.89	NA	−0.45 ± 12.33	NA
	T0–T2	−4.88 ± 3.98 *^C^	3.10 ± 10.14	NA	−0.63 ± 10.28	NA
CP	T0	NA	NA	NA	NA	NA
	T1	NA	NA	NA	NA	NA
	T2	NA	NA	NA	NA	NA
	T0–T1	NA	NA	NA	NA	NA
	T1–T2	NA	NA	NA	NA	NA
	T0–T2	NA	NA	NA	NA	NA

* Statistical significance of paired sample *t*-test (*p* < 0.05), A: T0–T1 intragroup comparisons within each subcategory, B: T1–T2 comparisons, C: T0–T2 comparisons, NA = not applicable.

**Table 7:** Comparisons of the manual and digital measurements in CLP patients (groups CLP-AP and CLP-PP) at T0 (pretreatment), T1 (6 months into the therapy and immediately prior to the operation) and T2 (1 year following the operation). N = sample size, ICC = intracorrelation coefficient, CI = confidence interval, ’ indicates the left side, NA = not applicable.

Distance/Angle	N	ICC (95% CI)		
		T0	T1	T2
P1-P2	9	0.994 [0.914, 0.998]	0.507 [0.105, 0.769]	NA
P1’-P2’	11	0.967 [0.920, 0.987]	0.940 [0.857, 0.976]	NA
CC’	20	0.982 [0.954, 0.993]	0.986 [0.946, 0.995]	0.991 [0.978, 0.996]
TT’	20	0.984 [0.960, 0.994]	0.970 [0.910, 0.989]	0.996 [0.991, 0.999]
CM	20	0.789 [0.367, 0.924]	0.797 [0.562, 0.914]	0.989 [0.972, 0.996]
C’M	20	0.869 [0.588, 0.953]	0.949 [0.879, 0.979]	0.336 [0.107, 0.669]
AM	20	0.995 [0.987, 0.998]	0.997 [0.993, 0.999]	0.972 [0.932, 0.989]
P1-TT’	9	0.981 [0.948, 0.993]	0.980 [0.951, 0.992]	NA
P2-TT’	9	0.954 [0.889, 0.981]	0.972 [0.933, 0.989]	NA
P1’-TT’	11	0.972 [0.932, 0.989]	0.991 [0.978, 0.996]	NA
P2’-TT’	11	0.776 [0.299, 0.921]	0.947 [0.872, 0.978]	NA
I-TT’	20	0.931 [0.712, 0.977]	0.976 [0.942, 0.990]	0.987 [0.968, 0.995]
I-CC’	20	0.974 [0.937, 0.990]	0.870 [0.707, 0.946]	0.990 [0.977, 0.996]
C-TT’	20	0.971 [0.871, 0.990]	0.989 [0.971, 0.996]	0.996 [0.991, 0.999]
C’-TT’	20	0.955 [0.794, 0.986]	0.918 [0.803, 0.967]	0.956 [0.891, 0.982]
P1-I	9	0.969 [0.924, 0.987]	0.974 [0.901, 0.991]	NA
P1’-I	11	0.770 [0.513, 0.902]	0.861 [0.679, 0.943]	NA
I-C	9	0.924 [0.822, 0.969]	0.811 [0.550, 0.923]	NA
I-C’	11	0.362 [−0.07, 0.685]	0.996 [0.991, 0.999]	NA
P1-C	9	0.948 [0.876, 0.979]	0.917 [0.758, 0.969]	NA
P2-C’	9	0.801 [0.570, 0.915]	0.951 [0.882, 0.980]	NA
P1’-C	11	0.366 [−0.07, 0.688]	0.964 [0.911, 0.985]	NA
P2’-C’	11	0.175 [−0.274, 0.563]	0.977 [0.930, 0.991]	NA
C-T	20	0.974 [0.925, 0.990]	0.989 [0.973, 0.996]	0.997 [0.991, 0.999]
C’-T’	20	0.935 [0.821, 0.974]	0.913 [0.797, 0.964]	0.957 [0.897, 0.983]
AKL (P1-I-C-T)	9	0.813 [0.227, 0.941]	0.774 [0.395, 0.914]	0.517 [0.124, 0.774]
AKL (P1’-I-C-T)	11	0.186 [−0.264, 0.571]	0 [−0.243, 0.330]	0.980 [0.952, 0.992]
AKL’ (P2-C’-T’)	9	0 [−0.09, 0.175]	0 [−0.088, 0.169]	0.699 [0.388, 0.868]
AKL’ (P2’-C’-T’)	11	0.082 [−0.113, 0.364]	0.0898 [−0.105, 0.371]	0.584 [0.209, 0.811]
MIA	20	0.882 [0.731, 0.951]	0.789 [0.547, 0.910]	0.897 [0.753, 0.958]
C-T-T’	20	0.986 [0.964, 0.994]	0.982 [0.954, 0.992]	0.977 [0.946, 0.991]
C’-T’-T’	20	0.892 [0.719, 0.958]	0.917 [0.806, 0.966]	0.985 [0.962, 0.993]
P1-C-T	9	0.934 [0.553, 0.981]	0.616 [0.162, 0.839]	NA
P2-C’-T’	9	0.908 [0.788, 0.962]	0.910 [0.788, 0.963]	NA
P1’-C-T	11	0.598 [0.115, 0.834]	0.960 [0.854, 0.986]	NA
P2’-C’-T’	11	0.115 [−0.331, 0.521]	0.920 [0.748, 0.970]	NA

## Data Availability

The data are available upon request from the corresponding author due to data privacy regulations of the J. W. Goethe University.

## Abbreviations

The following abbreviations are used in this manuscript:

UCLP	Unilateral Cleft Lip and Palate

## References

[1] Sader R. (2009). Lippen-Kiefer-Gaumen-Segelspalten. Pädiatrie Up2date.

[2] Tian H., Feng J., Li J., Ho T.V., Yuan Y., Liu Y., Brindopke F., Figueiredo J.C., Magee W., Sanchez-Lara P.A. (2017). Intraflagellar transport 88 (IFT88) is crucial for craniofacial development in mice and is a candidate gene for human cleft lip and palate. Hum. Mol. Genet..

[3] Shaw W. (2004). Global strategies to reduce the health care burden of craniofacial anomalies: Report of WHO meetings on international collaborative research on craniofacial anomalies. Cleft Palate Craniofac. J..

[4] Suri S., Disthaporn S., Atenafu E.G., Fisher D.M. (2012). Presurgical presentation of columellar features, nostril anatomy, and alveolar alignment in bilateral cleft lip and palate after infant orthopedics with and without nasoalveolar molding. Cleft Palate Craniofac. J..

[5] Liao Y.F., Cole T.J., Mars M. (2006). Hard palate repair timing and facial growth in unilateral cleft lip and palate: A longitudinal study. Cleft Palate Craniofac. J..

[6] Brand S., Blechschmidt A., Müller A., Sader R., Schwenzer-Zimmerer K., Zeilhofer H.F., Holsboer-Trachsler E. (2009). Psychosocial functioning and sleep patterns in children and adolescents with cleft lip and palate (CLP) compared with healthy controls. Cleft Palate Craniofac. J..

[7] Hotz M.M., Gnoinski W.M. (1979). Effects of early maxillary orthopaedics in coordination with delayed surgery for cleft lip and palate. J. Maxillofac. Surg..

[8] Schopf P. (2008). Werkstoffe, festsitzende Apparaturen, kieferorthopädische Therapie, interdisziplinäre Aspekte. Curriculum Kieferorthopädie/Peter Schopf.

[9] Habel A., Sell D., Mars M. (1996). Management of cleft lip and palate. Arch. Dis. Child..

[10] Gnoinski W., Nussbaumer H. (1976). Joint treatment of patients with clefts by orthodontists and logopedists. SSO Schweiz Monatsschr Zahnheilkd.

[11] Schwenzer N., Ehrenfeld M. (2010). Mund-Kiefer-Gesichtschirurgie.

[12] Kriens O., Bertzbach P. (1986). Model analysis of the maxilla in newborn infants with unilateral cheilognathopalatoschisis. Fortschr. Kieferorthop..

[13] Kozelj V. (1999). Changes produced by presurgical orthopedic treatment before cheiloplasty in cleft lip and palate patients. Cleft Palate Craniofac. J..

[14] Hotz M., Gnoinski W. (1977). Multidisziplinäre Betreuung von Patienten mit Lippen-Kiefer-Gaumenspalten: Vorläufige Ergebnisse. Inf. Orthodont. U. Kieferorthop..

[15] Bolter H. (1979). Oberkiefer- Alveolarbogenmasse bei LKG-Spaltträgern. Nach der Geburt und mit 5 Jahren: Eine Standortbestimmung der primären Behandlung in Zürich. Doctoral Dissertation.

[16] Opitz C., Kratzsch H. (1997). Oberkieferdimension bei Patienten mit ein- und doppelseitiger Lippen-Kiefer-Gaumen-Spalte. J. Orofac. Orthop/Fortschr. Kieferorthop..

[17] Dallaserra M., Pantoja T., Salazar J., Araya I., Yanine N., Villanueva J. (2022). Effectiveness of pre-surgical orthopedics on patients with cleft lip and palate: A systematic review and meta-analysis. J. Stomatol. Oral. Maxillofac. Surg..

[18] Shen C., Yao C.A., Magee W., Chai G., Zhang Y. (2015). Presurgical nasoalveolar molding for cleft lip and palate: The application of digitally designed molds. Plast Reconstr. Surg..

[19] Ambrosio E.C.P., Sforza C., De Menezes M., Carrara C.F.C., Machado M., Oliveira T.M. (2018). Post-surgical effects on the maxillary segments of children with oral clefts: New three-dimensional anthropometric analysis. J. Craniomaxillofac Surg..

[20] Saad M.S., Fata M., Farouk A., Habib A.M.A., Gad M., Tayel M.B., Marei M.K. (2020). Early Progressive Maxillary Changes with Nasoalveolar Molding: Randomized Controlled Clinical Trial. JDR Clin. Trans. Res..

[21] Sabri T.K. (2015). Dreidimensionale Auswertung des Wachstumsmechanismus bei Einseitiger Lippen-Kiefer-Gaumenspalte Nach Behandlung Mit der Modifizierten Trennplatte. Doctoral dissertation.

[22] Mazaheri M., Harding R.L., Cooper J.A., Meier J.A., Jones T.S. (1971). Changes in arch form and dimensions of cleft patients. Am. J. Orthod..

[23] Ashley-Montague M. (1934). The form and dimensions of the palate in the newborn. Int. J. Orthod..

[24] Sillman J.H. (1951). Serial study of good occlusion from birth to 12 years of age. Am. J. Orthod..

[25] García Abuabara A.N., Drescher D. (2010). Development on the Maxillary of Patients with a Unilateral Total Cleft with the Use of a Orthopaedic Plate. Two-dimensional Cast Analysis. Rev. Colomb. Investig. Odontol..

[26] Burgaz M.A., Cakan D.G., Yılmaz R.B.N. (2019). Three-dimensional evaluation of alveolar changes induced by nasoalveolar molding in infants with unilateral cleft lip and palate: A case-control study. Korean J. Orthod..

[27] Koo T.K., Li M.Y. (2016). A Guideline of Selecting and Reporting Intraclass Correlation Coefficients for Reliability Research. J. Chiropr. Med..

[28] Seckel N.G., van der Tweel I., Elema G.A., Specken T.F. (1995). Landmark positioning on maxilla of cleft lip and palate infant--a reality?. Cleft Palate Craniofac. J..

[29] Kahl B. (1990). Frühbehandlung von Kindern mit Lippen-Kiefer-Gaumen-Spalten—Kieferorthopädische Aspekte. Fortschritte Kieferorthopädie.

[30] Erkan M., Karaçay S., Atay A., Günay Y. (2013). A modified feeding plate for a newborn with cleft palate. Cleft Palate Craniofac. J..

[31] Nalabothu P., Benitez B.K., Dalstra M., Verna C., Mueller A.A. (2020). Three-Dimensional Morphological Changes of the True Cleft under Passive Presurgical Orthopaedics in Unilateral Cleft Lip and Palate: A Retrospective Cohort Study. J. Clin. Med..

[32] Grabowski R., Kopp H., Stahl F., Gundlach K.K. (2006). Presurgical orthopaedic treatment of newborns with clefts--functional treatment with long-term effects. J. Craniomaxillofac. Surg..

[33] Martelli D.R., Machado R.A., Swerts M.S., Rodrigues L.A., Aquino S.N., Martelli Júnior H. (2012). Non syndromic cleft lip and palate: Relationship between sex and clinical extension. Braz. J. Otorhinolaryngol..

[34] Sharif F., Mahmood F., Azhar M.J., Asif A., Zahid M., Muhammad N., Rehman I.U., Neil S.M. (2019). Incidence and management of cleft lip and palate in Pakistan. J. Pak. Med. Assoc..

[35] Kim S., Kim W.J., Oh C., Kim J.C. (2009). Cleft lip and Palate Incidence Among the Live Births in the Republic of Korea. JKMS.

[36] Neuschulz J., Schaefer I., Scheer M., Christ H., Braumann B. (2013). Maxillary reaction patterns identified by three-dimensional analysis of casts from infants with unilateral cleft lip and palate. J. Orofac. Orthop..

[37] Braumann B., Rosenhayn S.E., Bourauel C., Jäger A. (2001). Two- or three-dimensional cast analysis in patients with cleft lip and palate?. J. Orofac. Orthop..

[38] Hoffmannova E., Moslerová V., Dupej J., Borský J., Bejdová Š., Velemínská J. (2018). Three-dimensional development of the upper dental arch in unilateral cleft lip and palate patients after early neonatal cheiloplasty. Int. J. Pediatr. Otorhinolaryngol..

[39] Ambrosio E.C.P., Sforza C., De Menezes M., Gibelli D., Codari M., Carrara C.F.C., Machado M., Oliveira T.M. (2018). Longitudinal morphometric analysis of dental arch of children with cleft lip and palate: 3D stereophotogrammetry study. Oral. Surg. Oral. Med. Oral. Pathol. Oral. Radiol..

[40] Garland K., Coyle M., Foley T., Matic D. (2023). Ten-Year Cephalometric Comparison of Patients with Cleft Palate who Received Treatment with Active or Passive Pre-surgical Orthopedic Devices. Cleft Palate Craniofac. J..

[41] Kornbluth M., Campbell R.E., Daskalogiannakis J., Ross E.J., Glick P.H., Russell K.A., Doucet J.C., Hathaway R.R., Long R.E., Sitzman T.J. (2018). Active Presurgical Infant Orthopedics for Unilateral Cleft Lip and Palate: Intercenter Outcome Comparison of Latham, Modified McNeil, and Nasoalveolar Molding. Cleft Palate Craniofac. J..

[42] Matic D.B., Power S.M. (2008). The effects of gingivoperiosteoplasty following alveolar molding with a pin-retained Latham appliance versus secondary bone grafting on midfacial growth in patients with unilateral clefts. Plast. Reconstr. Surg..

[43] Cruz C. (2016). Presurgical Orthopedics Appliance: The Latham Technique. Oral. Maxillofac. Surg. Clin. N. Am..

[44] Power S.M., Matic D.B. (2009). Gingivoperiosteoplasty following alveolar molding with a Latham appliance versus secondary bone grafting: The effects on bone production and midfacial growth in patients with bilateral clefts. Plast. Reconstr. Surg..

[45] Winnand P., Ooms M., Heitzer M., Schaffrath K., Pankert T., Hölzle F., Raith S., Modabber A. (2024). Defining biomechanical principles in pre-surgical infant orthopedics in a real cleft finite element model. Int. J. Oral Maxillofac. Surg..

[46] Kiya K., Oyama T., Sone Y., Ishii N., Hosokawa K. (2015). A novel active intraoral appliance for presurgical orthopaedic treatment in patients with complete bilateral cleft lip and palate. J. Plast. Reconstr. Aesthet. Surg..

[47] Oosterkamp B.C., van Oort R.P., Dijkstra P.U., Stellingsma K., Bierman M.W., de Bont L.G. (2005). Effect of an intraoral retrusion plate on maxillary arch dimensions in complete bilateral cleft lip and palate patients. Cleft Palate Craniofac. J..

[48] Budak H., Yilmaz H.N. (2025). Evaluation of the Reliability of Facial Models Digitalized with Different Imaging Methods in Cleft Lip and Palate. Cleft Palate Craniofac. J..

[49] Luu N.S., Nikolcheva L.G., Retrouvey J.M., Flores-Mir C., El-Bialy T., Carey J.P., Major P.W. (2012). Linear measurements using virtual study models. Angle Orthod..

